# Adipose derived mesenchymal stem cell secretome formulation as a biotherapeutic to inhibit growth of drug resistant triple negative breast cancer

**DOI:** 10.1038/s41598-021-01878-z

**Published:** 2021-12-06

**Authors:** Ragima Nadesh, Krishnakumar N. Menon, Lalitha Biswas, Ullas Mony, K. Subramania Iyer, Sundeep Vijayaraghavan, Ajit Nambiar, Shantikumar Nair

**Affiliations:** 1 Amrita Centre for Nanosciences and Molecular Medicine, Amrita Vishwa Vidyapeetham, Kochi, Kerala 682041 India; 2 Amrita Institute of Medical Sciences and Research Centre, Amrita Vishwa Vidyapeetham, Kochi, Kerala 682041 India

**Keywords:** Biochemistry, Biological techniques, Biophysics, Cancer, Cell biology, Molecular biology, Stem cells

## Abstract

In the present study, a protocol was developed for processing of human adipose derived mesenchymal stem cell secretome formulation of varying concentration. Its molecular composition was evaluated, and its effectiveness in vitro using breast cancer cell lines, and in vivo in a nude mice breast cancer model was studied to determine its role in suppressing triple negative breast cancer in a dose dependent manner. Because the secretome could have value as an add-on therapy along with a current drug, the effectiveness of the secretome both in monotherapy and in combination therapy along with paclitaxel was evaluated. The results showed significant cell kill when exposed to the secretome above 20 mg/ml at which concentration there was no toxicity to normal cells. 70 mg/ml of SF showed 90 ± 10% apoptosis and significant decrease in CD44^+^/CD24^−^, MDR1+ and PDL-1+ cancer cells. In vivo, the tumor showed no growth after daily intra tumor injections at 50 mg/ml and 100 mg/ml doses whereas substantial tumor growth occurred after saline intra tumor injection. The study concludes that SF is a potential biotherapeutic for breast cancer and could be used initially as an add-on therapy to other standard of care to provide improved efficacy without other adverse effects.

## Introduction

While our previous work has focused on targeted therapy^1^ and drug delivery systems^2^ to find new solutions for cancer therapy, in this work we consider the use of an innovative biotherapeutic approach using the therapeutic potential of stem cell secretions and creating a new formulation with translational potential. In this study, we use in vitro and in vivo models of triple negative breast cancer^3^ (TNBC) lacking estrogen, progesterone and human epidermal growth factor 2 (HER2) receptors which is an aggressive form of breast cancer showing chemo^4,5^ and immune resistant phenotype with poor response to targeted therapies^6,7^ to evaluate the anti-cancer potential. Currently, treatment for TNBC includes neoadjuvant chemotherapy^8,9^ combined with optional PARP^10,11^ PDL-1^12^ inhibitors. Despite this, TNBC is likely to metastasize and recur^13^. Thus alternative strategies are essential for treating aggressive TNBC cells.

Biotherapeutics^14^, involving substances derived from living organisms, including proteins, enzymes, oligonucleotides, genes and cells for therapy is in trials as an alternate safe therapeutic to treat many illnesses. Considerable body of work has shown the value of antibody^15^ and immune checkpoint inhibitors^16^ for cancer therapy and are now approved therapeutics. There is strong evidence that stem cell therapy^17^ has substantial importance in regenerative medicine, with many clinical trials in progress^18–21^, although its use is still not FDA approved^22,23^ except for the case of hematopoietic stem cells^24^. The use of MSCs for stem cell therapy^25^ in cancer is quite far from clinical realization given the immune rejection potential for stem cell allografts due to the expression of foreign antigens on the stem cell surface^26^, as well as the possibility of teratogenesis^27^. However, stem cells can produce factors, such as, TNF-alpha related apoptosis inducing ligand (TRAIL)^28^ and IFN-beta^29^ both of which can suppress cancer. On the same token, stem cell produces factors that promote vascularization, metastasis and immunosuppression all of which can potentially cause the cancer to be promoted^30^. Thus stem cell therapy has to be viewed with caution. Importantly, stem cell paracrine secretions are highly sensitive to the cellular environment^31^, thus making the action of stem cells unpredictable^32^.

Nonetheless, use of stem cell secretome (the full set of molecular factors, including many proteins, secreted by the cell into the extracellular environment) can avoid allograft immune rejection^33^. Moreover, the secretome can be derived under controlled conditions in the lab and perhaps even engineered in a bioreactor^34^ to contain favourable anti-cancer factors, thus making it a more reliable translational approach as a bio-therapeutic for cancer. There have been few studies on the effect of stem cell conditioned media (meaning here media that has been exposed to stem cells or conditioned by stem cells), as obtained from normal stem cell cultures, on cancer cells under in vitro conditions^35–50^. The results reported by Schneider et al.^37^ and Kanehiraet al.^38^, showed that the conditioned media derived from mesenchymal stem cells isolated from a healthy physiological source (lungs, femur head and bone marrow of normal donors) have the ability to inhibit cancer cell growth. Martin et al.^39^ and Gauthaman et al.^35^ reported that it significantly induced apoptosis in cancer cells with aggressive phenotype. On the other hand, the stem cell conditioned media has also shown the opposite effect in some cases, namely promotion of cancer^46–50^. This variability may be attributed to how the conditioned media is prepared. Questions that arise are: Is the concentration of secretome adequate in the media when they are exposed to stem cells? Do nutrients in the media get exhausted after exposure to stem cells for a long period? If the secretome concentration is too small cancer may not be inhibited. Further, if the media is too exhausted, this factor can inhibit cancer cell proliferation irrespective of the effect of secretome factors. Thus, development of a stem cell secretome formulation which can reproducibly demonstrate the suppression of cancer is essential.

In the present study, we develop a protocol for the use of human adipose derived mesenchymal stem cell (MSC) secretome to create a formulation with well-defined secretome concentration, evaluate its molecular composition, and explore its effectiveness, in vitro and in vivo, in suppressing breast cancer in a dose dependent manner. We have selected MSCs because previous studies use MSCs as they have clinical translational potential and can be readily derived from adipose tissue^41,43^ and expanded. To further prove its therapeutic effect, we evaluated its effectiveness in monotherapy and in combination therapy along with paclitaxel. This allows for consideration of the secretome formulation to be used as an add-on therapy to standard chemotherapy. This is the first such comprehensive study of MSC secretome as a potential anti-cancer bio-therapeutic against TNBCs.

## Results

### Characterisation of MSC secretome using physical and biochemical characterisation methods

**Figure 1:**
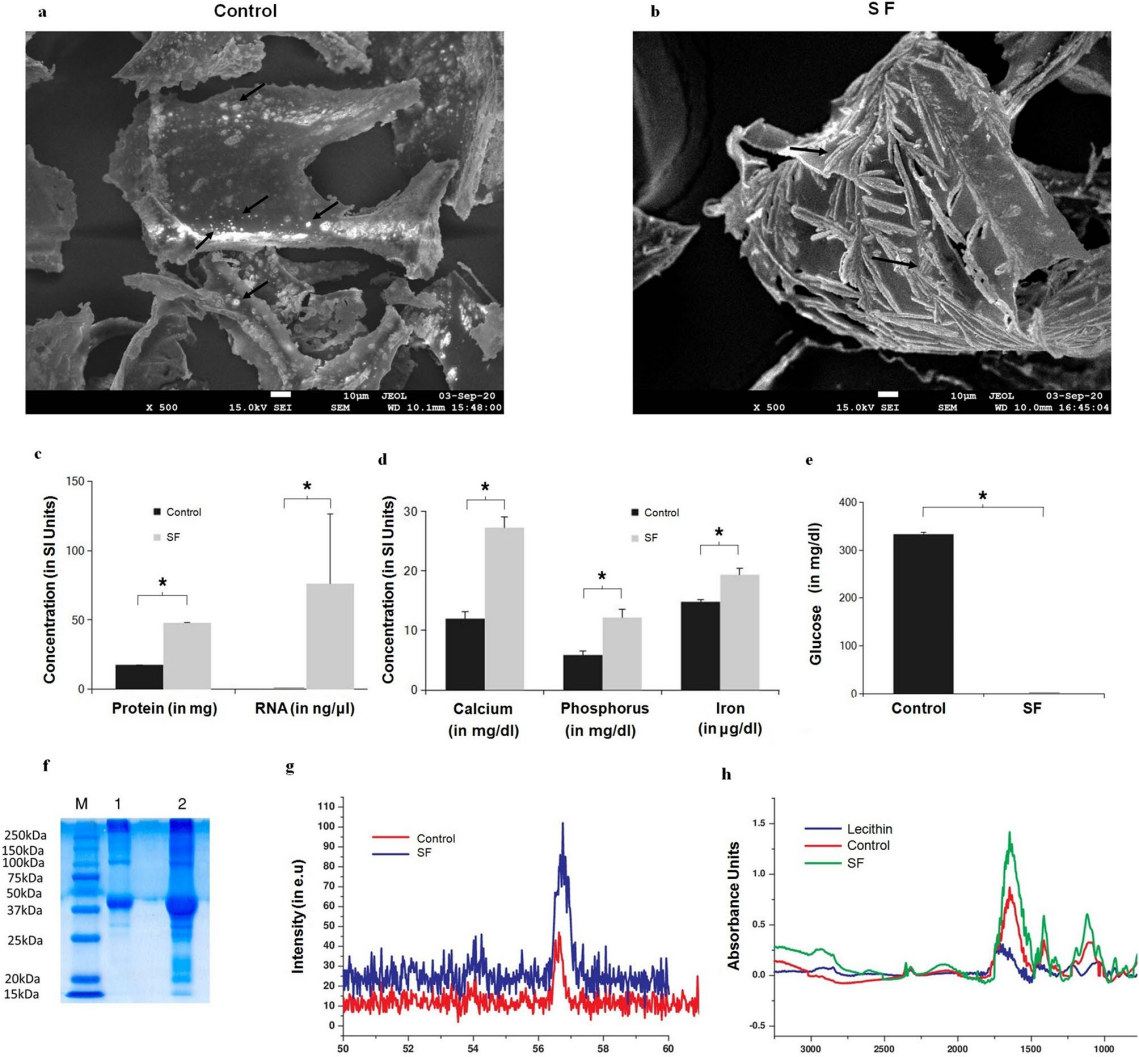
Characterization of freeze dried adipose derived stem cell secretome. (**a**) FESEM image of freeze dried medium alone (that is without secretome), black arrows shows crystalline particles, (**b**) FESEM image of freeze dried secretome, black arrows shows acicular crystalline flakes suggesting protein and/or RNA content in SF; (**c**) Graphical plots of protein (n = 3) and RNA (n = 3) quantified showing higher concentrations of proteins, and also higher levels of RNA in SF; (**d**) Graphical plots of minerals profiled of Calcium, phosphorus and iron again showing increased content in SF, (**e**) estimated glucose levels showing negligible glucose in SF; (**f**) Scanned Image of SDS Page (M—Marker proteins, Lane 1- control and Lane 2- freeze dried secretome) confirming more numerous and higher protein content in SF compared to control; (**g**) Graphical plots of XRD analysis with the main peak confirming higher mineral content. (**h**) Graphical plots of FTIR analysis, with the peaks indicating higher lipid levels in SF. All graphical plots are mean ± SD values and differences between control and treatment concentrations were considered statistically significant for *p* value < 0.05 and is represented by (*).

To understand the structural features in stem cell secretome upon freeze drying, field emission scanning electron microscope (FESEM) imaging was done. It is evident from Fig. 1a that compared to control (the freeze dried chemically defined media) the secretome displayed acicular features typical of crystalline precipitates that could be attributed to proteins and RNA in freeze dried secretome (Fig. 1b). To quantify biochemical components in secretome, we performed biochemical and physical characterisation of freeze dried secretome. Results revealed more than twice the amount of proteins (Fig. 1c) and (66.45 ± 16.47 ng/ul RNA in 100 mg of secretome compared to almost none in control (Fig. 1c). Among the minerals quantified calcium, phosphorous and iron content was 15, 7 and 5 units more in secretome compared to control (Fig. 1d). Glucose level in secretome was 300 times less than control (Fig. 1e), and SDS PAGE data (Fig. 1f) showed higher concentration of proteins in secretome compared to control. Further, freeze dried secretome had double the amount of urea compared to control (Supplementary information). X-ray diffraction results in Fig. 1g show a prominent increase in the calcite peak at 54 and 57, confirming increased mineral content in SF. Likewise, the Fourier Transform Infrared Spectroscopy (FTIR) results in Fig. 1h show peak at 3000–2700 cm^−1^ representing signals associated with presence of lipid hydrocarbon content and peaks at 1000–1700 cm^−1^ representing esters and acidic carbon bonds, (C=O, and C–O) in lipids.

From these data, we conclude that, freeze dried secretome powder is rich in protein, lipids and RNA content along with Ca, Fe and P present as trace elements compared to protein and RNA that are the major biomolecules in secretome.

### Effect of adipose derived stem cell secretome formulation (SF) on TNBCs

**Figure 2:**
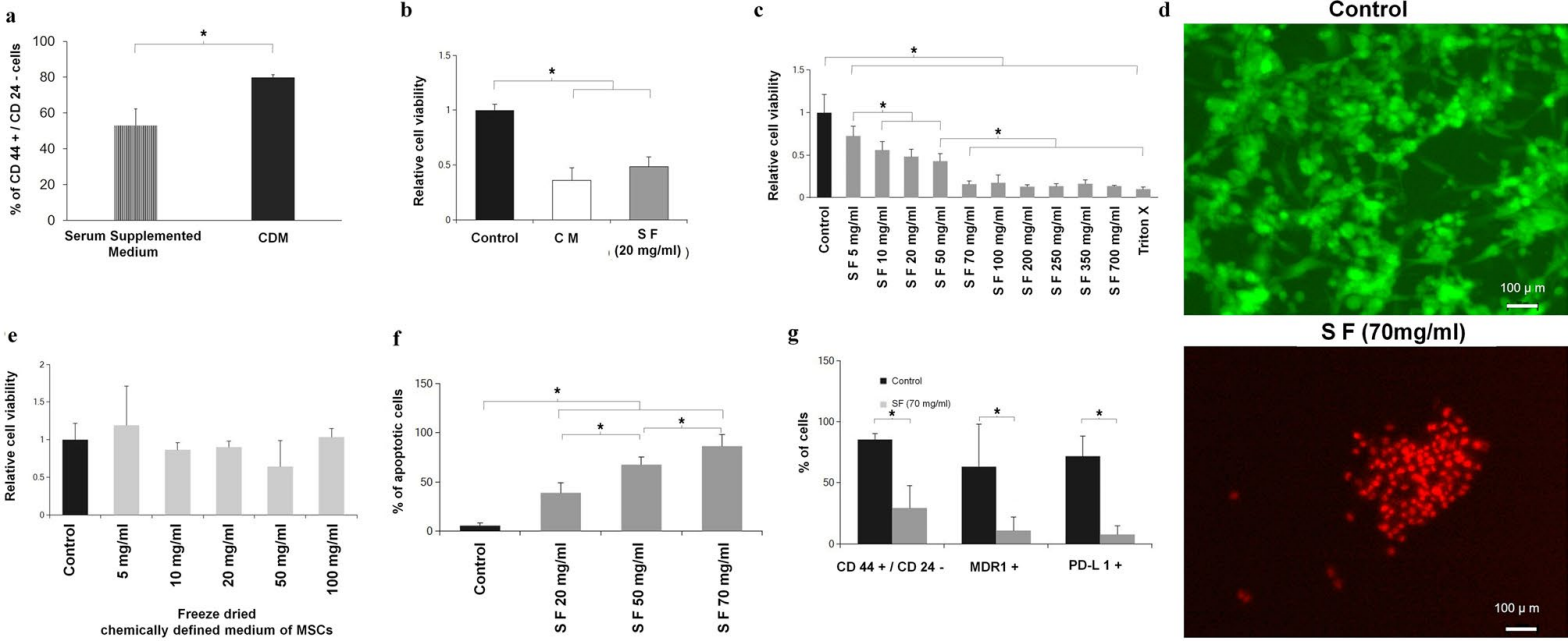
Adipose derived stem cell secretome on TNBC viability, apoptosis and molecular markers. (**a**) Flow cytometry results of TNBCs in serum supplemented medium and chemically defined medium with CD44^+^/CD24^−^ breast cancer stem cell phenotype after 48 h. The chemically defined medium increased marker concentration because of the presence of insulin, hydrocortisone and epidermal growth factor that are known to contribute to colony formation characteristic feature of cancer stem cells^51^. (**b**) Graphical plot of cell viability comparison between control (medium without MSC exposure), MSC conditioned medium (without lyophilization), and SF. SF at the low concentration of 20 mg/ml is equivalent to the MSC conditioned media results. (**c**) Dose dependent effect of SF on TNBC viability at 5, 10, 20, 50, 70, 100, 200, 250, 350 and 700 mg/ml. Above a dose of 50 mg/ml SF has substantial cell kill. (**d**) Effect of secretome free media showing that lyophilization of the media alone does not affect cell viability. Green color shows calcein binding to actin filaments in live cells. (**e**) Fluorescence image of live dead staining of TNBCs treated with 70 mg/ml SF for 48 h showing cell kill. Red color shows ethidium homodimer binding to nucleic acid in dead cells. (**f**) Apoptosis of cells treated with varying concentrations of SF for 48 h evaluated by Annexin-V–PI staining using flow cytometry showing an increase in cancer cell apoptosis. (**g**) Histogram of flow cytometry results showing the effect of 70 mg/ml SF on CD44^+^/CD24^−^ breast cancer stem cells (CSCs), multi drug resistance protein 1 (MDR1) and programmed death ligand-1(PD-L1) expressing cells. All markers are substantially decreased by SF. See text for discussion. All graphical plots are mean ± SD values and differences between control and treatment concentrations were considered statistically significant for *p* value < 0.05 and is represented by (*).

The formulation base, namely, the chemical defined medium (CDM) for TNBC cells, increased the population of cancer stem cells (CD44^+^/CD24^−^ phenotype) compared to DMEM, which is consistent with the literature^51^ (Fig. 2a). Stem cell conditioned media represented as CM in Fig. 2b and about 20 mg/ml concentration of SF (Supplementary information) gave similar results for cell viability (Fig. 2b). The key data of Fig. 2c clearly and convincingly shows a concentration dependent decrease in cell viability. The IC50 value was found by first plotting the data on a log concentration scale, see Supplementary Data Fig. S2A. IC50 was found using non-lineat regression fit using Prizm software. The IC 50 was 10.54 mg/ml with a range from 6.534 to 15.29 mg/ml. The SF treatment beyond 70 mg/ml concentration was found to be statistically indistinguishable from Triton X100 that cause complete cell lysis (Fig. 2c). It is important to point out that this concentration dependence was not the result of lyophilization. When the chemically defined media was lyophilized without exposure to stem cells there was no decrease in cell viability (Fig. 2e). Ethidium homodimer uptake by 70 mg/ml SF treated TNBCs confirms cell death compared to control cells that showed characteristic cytoskeletal feature of live cells using calcein stain (Fig. 2d).

### SF induce apoptosis in TNBCs

Based on the significant reduction in cell viability observed (Fig. 2b, c), we evaluated whether the cells are undergoing apoptosis. Annexin-V/PI staining of cells (Fig. 2f) showed a dose dependent apoptotic effect which was statistically significant compared to control (CDM) when treated with increasing concentrations of SF.

### Effect of SF on molecular markers

Since TNBC population express increased number of cancer stem cells with CD44^+^/CD24^−^ (Fig. 2a), we evaluated the levels of these markers in the TNBC population following SF treatment. It was observed that cancer stem cells with CD44^+^/CD24^−^ markers was substantially reduced (Fig. 2g) despite the increase in the presence of cancer stem cell population with this phenotype in chemically defined medium of TNBC cells (Fig. 2a). Clearly SF had a chilling effect on cancer stem cells (Fig. 2g). MDR1^+^ (multi-drug resistance protein 1) and PD-L1^+^ (programmed death ligand 1) phenotype was substantially reduced following treatment with 70 mg/ml SF.

### Viability of TNBC spheroids exposed to secretome formulation (SF-3D)

**Figure 3:**
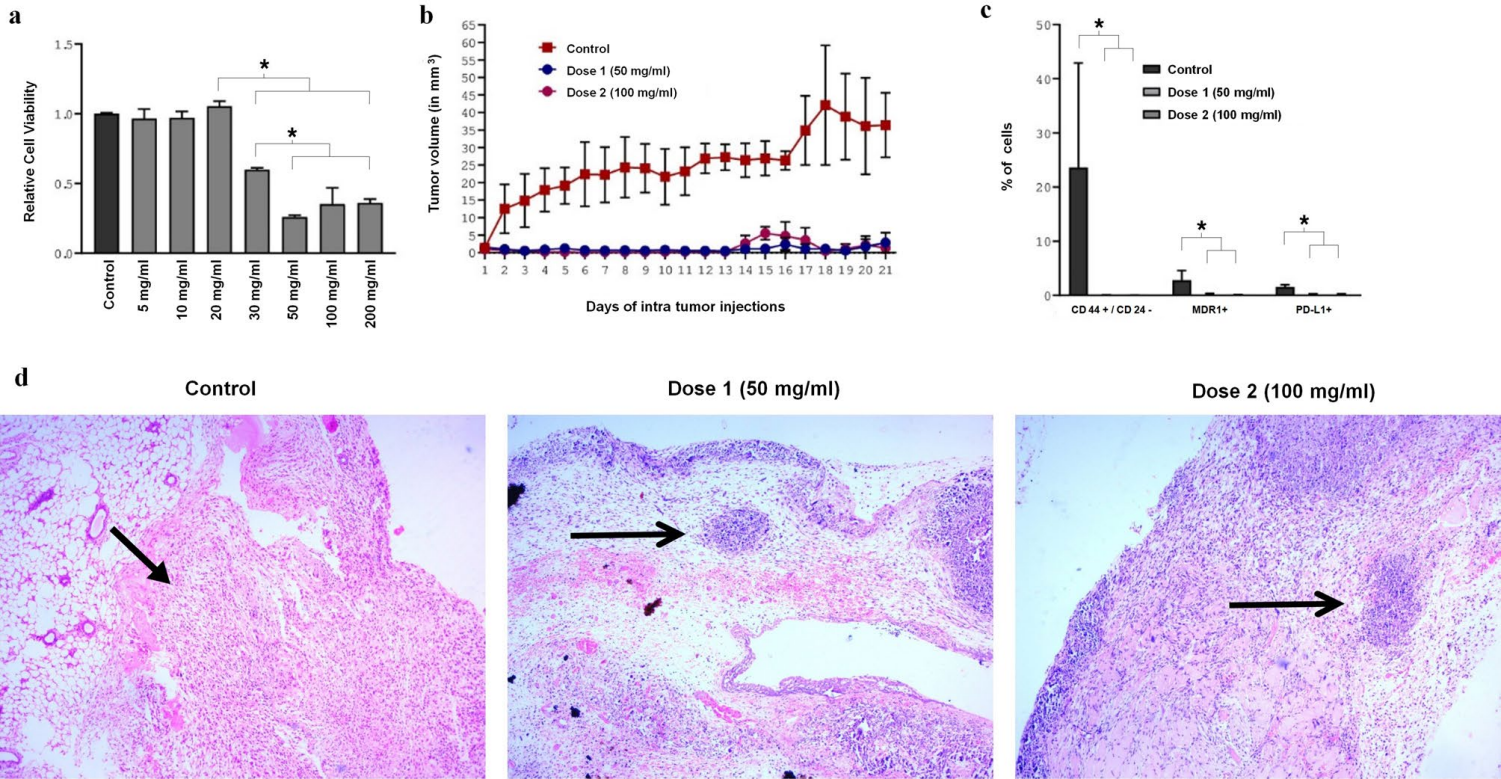
3D culture and in vivo results on tumor viability using secretome formulation. (**a**) MTT results of dose dependent effect of SF (freeze dried secretome in Mammocult control) on TNBC spheroids after 48 h showing MIC value above 30 mg/ml. (**b**) Graphical plot of tumor volumes of each animals in three groups (control Ringer’s lactate saline, dose1-50 mg/ml and dose2-100 mg/ml); n = 6 for each group. Intratumoral inection was given at tumor size of 1 mm3 and further tumor growth was absent only when SF was injected. (**c**) Results of flow cytometry to analyse CD44^+^/CD24^−^ breast cancer stem cells, multi drug resistance protein 1 (MDR1), programmed death ligand-1(PD-L1) expressing cells in xenograft tissue. Drastic decrease in all markers was found in treated cancer tissue. (**d**) Representative images of hematoxylin and eosin stained tumor from each group showing blue stained tumor necrotic regions (see arrows pointing to these regions) in the treated cancer tissue samples. Surrounding the necrotic regions are regions cleared of cancer tissue and populated by murine cells. The pink stained tumor stroma is not visible in these regions. In the control, that is saline injected, the arrow points to cancer tissue with extensive pink-stained cancer stroma. The stroma is filled with cancer cells. All graphical plots are mean ± SD values and differences between control and treatment concentrations were considered statistically significant for *p* value < 0.05 and is represented by (*).

Figure 3a is the dose dependent effect of the SF on tumor spheroids. As in the 2D case, there was a strong dose-dependent decrease of cell viability. Under 3D spheroid culture conditions IC50 value of SF was calculated using Prizm software with the concentration on a log scale as for the data in Fig. 2c. The analytical plot is shown in Supplementary Fig. S2B. The IC50 for the 3D case was 32.57 mg/ml with the range from 27.88 to 47.54 mg/ml.

### In vivo results in TNBC xenograft model

In all animals a distinct tumor mass of 1–2 mm^3^ was clearly visible and palpable in the range of 7–9 days. In a set of 6 control animals, only the saline without secretome was injected once the tumor mass was detected and in every single case the tumor mass continued to grow to a size on the order of 40 mm^3^, see Fig. 3b (the red curve). However, when SF was injected at the same time period after detection of tumor mass, no further growth of the tumor could be detected in every case in 12 animals at two doses (Fig. 3b) confirming that SF inhibited tumor growth.

The histopathology analysis (Fig. 3c) of excised tumor specimens confirmed that TNBC tumor cells in treatment groups were cleared with evidence of necrotic debris and infiltrated murine cells. The arrow in the treatment group point to the rounded necrotic mass surrounded by spots which are the infiltrated murine cells without the pink tumor stroma. The arrow in the control (saline control injection) point to the tumor mass where the stroma stains pink. The tumor appears to have been cleared by the treatment to create the necrotic patches surrounded by new murine cells. From H&E results (Fig. 3c) and T2 weighted MRI images (Supplement data of Fig. S1), it is evident that the remnant tissue in the MRI images of treatment groups is a mass of murine cells which migrated into the xenograft tumor stroma during the treatment time which appeared as thick fibrotic tissue. Flow cytometry analysis of tumor samples (Fig. 3d) further confirmed that SF at 50 mg/ml eliminated 99.76 ± 0.05% CD44^+^/CD24^−^ cells, 92.80 ± 0.26% MDR1 and 78.57 ± 0.11% PDL-1 positive cells from the tumor stroma. 100 mg/ml SF eliminated CD44^+^/CD24^−^, MDR1^+^, PDL-1^+^ cells respectively by 99.88 ± 0.05%, 91.72 ± 0.05% and 80.51 ± 0.1%.

### SF inhibit growth of paclitaxel resistant TNBCs

**Figure 4:**
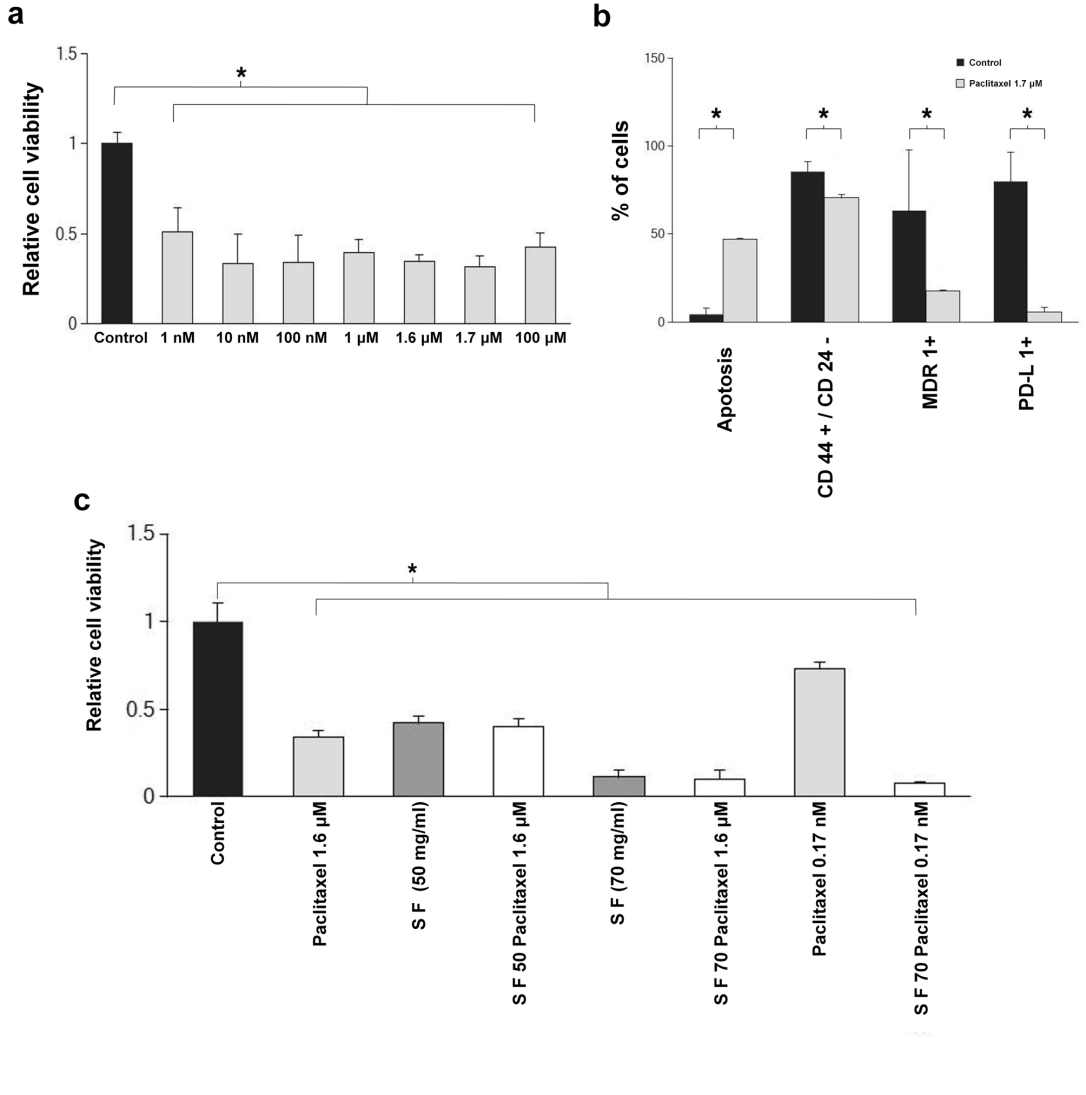
Influence of paclitaxel and SF in chemically defined media of breast cancer cells. (**a**) Graphical plot of MTT assay and comparison of relative cell viability between different doses of paclitaxel ranging from 1 nM to 100 µM for 48 h with control cells in chemically defined media of TNBCs. The cell viability is unchanged over a wide range of concentrations indicating cancer cells are resistant towards paclitaxel. (**b**) Flow cytometry results of Annexin V/PI staining, analysis of CD44^+^/CD24^−^ breast cancer stem cells (CSCs), multi drug resistance protein 1 (MDR1), programmed death ligand-1(PD-L1) expressing TNBCs treated with 1.7 µM paclitaxel after 48 h compared to control cells in chemically defined media of TNBCs. Though paclitaxel reduces MDR1+ and PDL1+ markers the cancer cells are still resistant to paclitaxel. The reduction in the markers is not nearly as much as the reduction caused by SF (see Fig. 2g). (**c**) Histogram plot of MTT results of SF 50 mg/ml, SF 70 mg/ml, Paclitaxel (1.6 µM, and 0.17 nM) and combinations of 50 mg/ml with 1.6 µM, 70 mg/ml with 1.6 µM and 0.17 nM on TNBCs after 48 h in 2D culture conditions compared to control. All data with SF of 70 mg/ml was statistically lower than when SF 50 mg/ml was used and resulted in significant decrease in the population of the resistance cancer cells. Such additions could be an add-on therapy to chemotherapy. All graphical plots are mean ± SD values and differences between control and treatment concentrations were considered statistically significant for *p* value < 0.05 and is represented by (*).

To further assess whether SF is synergistic with the conventional chemo-drug paclitaxel, we evaluated cell viability of TNBCs in a chemically defined media of breast cancer cells in 2D culture following treatment with different amounts of paclitaxel and in combination with SF. The fact that the cells were resistant to paclitaxel is evidenced by the fact that there was no effect of paclitaxel beyond a concentration of 1 nM (Fig. 4a). The influence on molecular markers of TNBCs in 2D culture with and without paclitaxel was evaluated (Fig. 4b). While paclitaxel increased cell apoptosis and decreased marker levels at a concentration of 1.7 µM (equivalent to 1.5 µg/ml), its impact was not nearly as much as the influence of SF on the same markers (Fig. 2g).

To further assess whether SF is synergistic with the conventional chemo-drug paclitaxel, we evaluated cell viability of TNBCs in a chemically defined media of breast cancer cells in 2D culture following treatment with different amounts of paclitaxel and in combination with SF (Fig. 4c). The main takeaway from this figure is that SF synergizes with paclitaxel only above 50 mg/ml SF concentration. One can note, especially from Fig. 4a that even at 100 µM TNBCs are resistant to treatment by paclitaxel alone. The equivalent blood concentration for human iv clinical dose (175 mg/m^2^) is about 60–70 µM. Substantial cancer cell kill can be achieved by SF as add-on therapy at SF blood concentration of 70 mg/ml. In fact SF alone at 70 mg/ml is superior to any of the paclitaxel doses.

### Effect of SF and paclitaxel on TNBC spheroids

**Figure 5:**
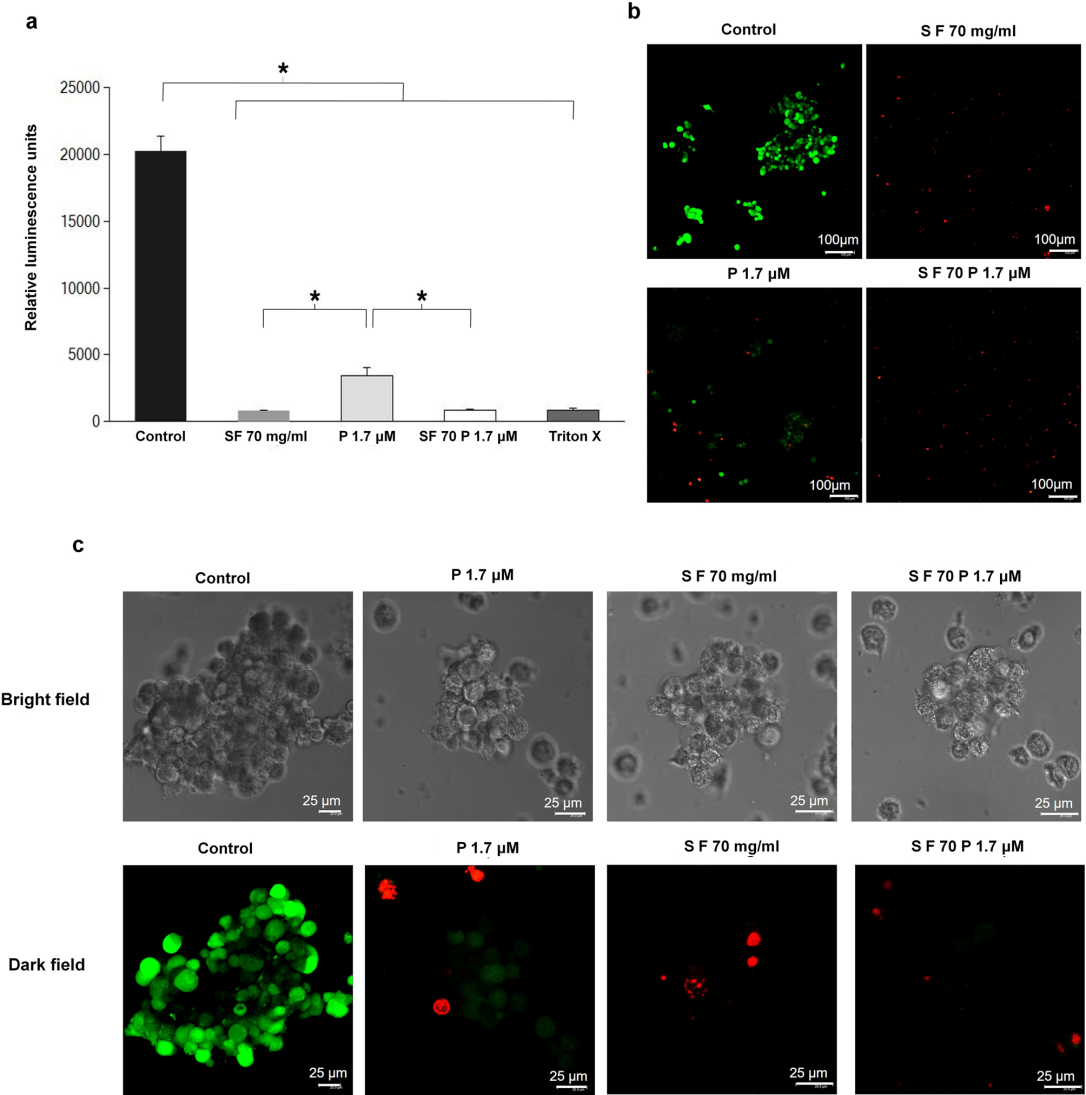
Cell survival using microscopy in 3D culture. (**a**) and (**b**) virtually complete death using cell titre glow assay in 3D culture with respect to control when SF is used. The green color intensity is proportional to the number of viable cells. (**c**) bright and dark field confocal Live cell images of TNBC spheroids after 48 h exposure to SF 70 mg/ml, P1 1.7 µM and combination of both, scale bar 100 µm confirming the results of (**a**) and (**b**). All graphical plots are mean ± SD values and differences between control and treatment concentrations were considered statistically significant for *p* value < 0.05 and is represented by (*). Green represents the live cells and the dead cells are shown red in color (see caption in Fig. 3).

To confirm the efficacy of SF, we compared its toxic effect on TNBC spheroids with paclitaxel combination. Results of spheroid cell titre glow assay (Fig. 5a) suggest that paclitaxel dose (1.7 µM) when combined with 70 mg/ml SF was found to be as effective as Triton X. Similar result was obtained with SF70 alone. Live-dead staining of cancer spheroids confirmed significant cell death (Fig. 5b, c) in 70 mg/ml SF.

## Discussion

One of the major hurdles in cancer therapy is the lack of appropriate treatments that would annihilate the cancer cells to increase the survival of patients. Thus, combinations of various chemicals were used based on the overall molecular phenotype of the type of cancer cells. Therefore, any new strategy that would help in the treatment of any aggressive form of cancer will be welcoming. One such aggressive form of cancer is TNBCs. Current treatment option for treating TNBC’s cantered around paclitaxel^6,7^ and neo-adjuvant therapy^8,9^. Over a decade, it is becoming clear that stem cell conditioned medium containing secretome may be a good therapeutic in treating cancer^35–45^. Thus, we here show the functional and biochemical characterization of MSC secretome and its effectiveness in inducing apoptosis in TNBC cells.

Though previous studies had characterised and identified proteins^52–54^ in the secretome, we have prepared the stem cell secretome in a GMP compliant^55^ FDA approved stem cell media and quantified all the major biomolecules in the freeze dried powder (Fig. 1). On the same note, lipid spectral characteristics seen in Fig. 1h is indicative of anti-cancerous nature as suggested by Brossa et al.^56^. Mineral components profiled in Fig. 1 could be the crystalline particles seen in control (Fig. 1a) and flakes could be formed from protein (growth factor supplements in the chemically defined medium of stem cells) FESEM image (Fig. 1a) and (carbohydrate) glucose quantified in Fig. 1f. Ca, P and Fe are essential elements constituted as inorganic nutrient supplements in such media. Fe is a key element in cancer cell metabolism and Ca and P are thought to play a beneficial role in cancer. These minerals present in secretome and not present in the form of crystals on flakes in Fig. 1b could be due to the presence of metallo-proteins in secretome. Protein and RNA are major components in freeze dried secretome and could be forming the needle like structures in Fig. 1b. Exosomes^57^ present in the secretome could not be definitively identified in control, Fig. 1a.

Although stem cell secretome was prepared by many by conditioning stem cells in a growth medium for a specific time^35–50,52–54^, the process if not controlled^52^ and balanced with respect to incubation conditions could lead to nutrient deprivation resulting in cell death or growth arrest^35–45^. As a result, contradicting results have been reported^46–50^ and could be attributed to the variability in preparation and processing methods. Thus, to address this issue, we designed our first experiment to address the issue of whether the anti-cancer effects reported^35–45^ was the outcome of using stem cell conditioned media that is deprived of nutrients or due to the secretome. Towards this, freeze dried secretome were supplemented with nutrients in chemically defined media of TNBCs (CDM). The media alone significantly increased population of cancer stem cells (CD44^+^/CD24^−^ phenotype) in TNBCs (Fig 2a) likely due to the presence of insulin, hydrocortisone and epidermal growth factor that are known to contribute to colony formation characteristic feature of cancer stem cells^51^. However, the same markers were substantially decreased when the same media contained secretome (Fig. 2g). For the first time, we show a dose dependent cytotoxic effect of SF which is similar to the anti-cancer effect of secretome reported previously^35–45^. At 20 mg/ml, the effect on cancer cell death was 50% (Fig. 2b). However, at higher concentrations of SF significant cancer cell death could be observed (Fig. 2c) up to 80%.

It is clear from Fig. 2e with different amounts of lyophilized powder of the chemically defined media of MSCs added to the chemical defined media of TNBCs without exposure to MSC cells (secretome free) that the cell viability shown in Fig. 2e was not affected compared to control. This suggested there was no effect of changes in the salt concentrations in the chemically defined medium affecting cellular homeostasis. Further, SF dose dependent decrease in cell viability (Fig. 2c) corresponded with in Fig. 2f showing cellular apoptosis, which suggested that decreased cell viability observed, is indeed due to the result of apoptosis. Since the cell line we used belonged to mesenchymal stem like and mesenchymal like category (MDA-MB-231) among the six different TNBCs due to heterogeneity, we checked for stemness markers CD44^+^/CD24^−^ and MDR1+^4,5^ (multi drug resistance protein 1), and also PDL1+^6,58^ (Programmed Death Ligand 1) as these population of cells can be potentially harmful with respect to invasion and aggressiveness. To our surprise, the percentage of total CD44^+^/CD24^−^, MDR1+ and PD-L1+ TNBCs following treatment with SF was brought down dramatically (Fig. 2g) suggesting the significance of secretome medium in the removal of these aggressive phenotypes from the TNBC population. To further verify our findings we checked the effect of SF on xenograft models of TNBC’s.

Tumor microenvironment is known to play a critical role in the response to therapy^59^ and hence to confirm the anti-cancer potential, tumor spheroids were treated with SF. Results in Fig. 3a and supplementary Fig. S2B showed that IC50 value of SF in 3D culture was 32.57 mg/ml three times of the IC50 value in 2D culture shown in Fig. 2c and Supplementary Fig. S2A, the latter being only 10.54 mg/ml SF. Thus there was found evidence indicating greater resistance of spheroids to secretome formulation treatment. It was interesting to note that 50 mg/ml SF in 3D culture (Fig. 3a) that had similar effect to 20 mg/ml SF in 2D (Fig. 2c) signifying a difference in response associated with more stem like characteristics^59^ and metabolic deregulation^60^.

SF was administered into the tumor for 21 consecutive days in the light of treatment duration previously reported by others^10–12,41^. Thus, the calculated equivalent dose corresponding to once a day intra-tumor administration of 10X secretome is found to be 200 mg/ml^41^. Thus, we treated tumor with 50 and 100 mg/ml SF led to undetectable TNBCs from 1 mm^3^ tumor stroma (Fig. 3b). As shown in Fig. 3b, c, significant suppression of overall tumor growth and CD44^+^/CD24^−^, MDR1+^3–5^ and PD-L1+^6,58^ cell types that are involved in stemness and invasion was observed indicating the significance of SF in effectively controlling TNBC growth. Indeed, eradication of these populations has immense value in developing therapies for cancer as cancer stem cells very much drive tumorigenesis, metastasis and therapeutic resistance^3–7^. The histopathology data of Fig. 3d clearly showed necrotic tumor patches with murine cells in the vicinity that developed after SF in vivo treatment.

Figure 4a shows that there was no significant difference between 1 nM and 100 µM compared to the reported 100% effective concentration of 10 nM^61^. This is evidence of paclitaxel resistance. Interestingly, the extent of reduction of CD44^+^/CD24^−^ breast cancer stem cells, multi drug resistance protein 1 (MDR1), programmed death ligand-1(PD-L1) expressing cells were far less than that due to SF seen in Fig. 2g. It is to be noted that many reports indicate the use of taxane based therapies for TNBC type^9^ to overcome the drug resistance mechanisms associated with these cell types^3,9^. Thus, we compared the effect of paclitaxel with SF by treating TNBCs with SF and paclitaxel near to its IC50 value and beyond singly or in combination (Fig. 4). Although, at the IC_50_ value of paclitaxel we observed significant cell death, a significant population of TNBC’s were resistant to paclitaxel and that SF medium alone at 70 mg induced significantly more death of TNBC’s than paclitaxel (Fig. 4a–c).

It has been reported that tumor micro environment plays significant role in determining the therapeutic efficacy of paclitaxel^3,59^. Thus, we verified the effect of SF and paclitaxel in a 3D environment to find its influence on the observed cell death. Using the developed 3D cultures of TNBC (Fig. 5), we found similar pattern of cell death as observed in our 2D and in vivo studies confirming the effect and significance of SF medium in inducing cell death. Nonetheless, Figs. 4c and 5 confirms prospective use of SF 70 mg/ml in combination with lower doses of paclitaxel. This clearly demonstrate that a combination of MSC secretome with lower doses of paclitaxel can be used to minimise toxic side effects of using higher doses of paclitaxel as previously reported^62^ in the treatment of highly chemo resistant TNBC’s in clinics.

All the results discussed in Figs. 1, 2, 3, 4 and 5 suggest the prospective therapeutic potential of SF for clinical management of highly chemo resistant cancer types. Perhaps SF formulation could also favour efficacy of paclitaxel at lower dose which is otherwise highly neuro-toxic when administered at higher doses^62^. It would be interesting to fractionate or separate each of the biomolecule in Fig. 1 to further study the contribution of them towards the anti-cancer effects discussed. In conclusion our study clearly illustrates that SF is effective in suppressing tumor growth and inducing TNBC cell apoptosis in a manner efficient than paclitaxel. Importantly, cells with PDL1+, CD44^+^/CD24^−^, MDR1+ phenotypes that are potentially harmful with respect to invasion and aggressiveness could significantly be eliminated from TNBC population. Moreover, combination of SF with paclitaxel could be used to reduce the toxic effect of paclitaxel and add value to the finding. In addition, targeting the genes associated with cancer progression and survival following SF treatment could improve the treatment outcome. Further in-depth gene expression studies are required to apprehend the detailed molecular path ways associated with apoptotic mechanisms that are responsible for the anti-cancer potential of stem cell secretome. Protein and RNA characterisation of the secretome would also help to identify molecules responsible for nucleic acid degradation seen in spheroids and mechanisms involved in immune cell infiltration into tumor stroma. Based on our results, we envisage the potential of SF in combination therapies for the treatment of aggressive forms of TNBC.

## Materials and methods

### TNBC cell line and culture conditions

MDA-MB-231 (TNBC type) breast cancer cells was procured with certificate of authentication from National Centre for Cell Science (India) and were cultured in 10% FBS and 1% antibiotic (Gibco, USA) supplemented DMEM high glucose (Invitrogen, USA). This is referred to here as DMEM. For all in vitro studies a chemically defined serum free medium (MEGM, Lonza, Switzerland) was used as control, referred to here as CDM so as to avoid the interferences from proteins in the serum supplemented media (DMEM).

### Isolation of MSCs and characterization using flow cytometry

MSCs were isolated from abdominal lipoaspirates. Briefly, lipoaspirates were collected from healthy female patients undergoing liposuction procedure after obtaining approval from Institutional ethics Committee at Amrita Institute of Medical sciences and Research Centre in accordance with the basic guidelines and regulations. All patient lipoaspirate samples were collected after obtaining informed consent based on IEC specifications. Subsequently, lipoaspirate was washed in PBS containing antibiotics and centrifuged at 200 g for 5 min. After 2–3 washes, adipose tissue was digested with equal volume of 0.2% of 290 Units/mg collagenase Type I (Gibco, USA) for 1 h at 37 °C with intermittent shaking. Further, the digested tissue was centrifuged at 300 g for 10 min and the pellet obtained was washed in growth medium, [10% MSC specific FBS (Gibco, USA) and 1% of 10,000 Units/ml antibiotics (Gibco, USA) supplemented IMDM (Invitrogen, USA)]. Cell pellet obtained after centrifuging at 270 g for 5 min was maintained in 2D culture, characterised for MSC markers before secretome preparation.

### Preparation of MSC secretome formulation (SF)

Passage 3–7 MSCs were seeded in T75 flasks and when 80–90% confluent, cells were incubated for 48 h in 15 ml of chemically defined MSC specific medium (Gibco, USA) supplemented with 1% (200 mM) glutamine (Thermo Fisher, USA) and 1% antibiotics (Anti-Anti, Gibco, USA) for preparing the stem cell conditioned media. Media collected was centrifuged at 270 g for 5 min, frozen at -20 °C, freeze dried (Fisher Alpha 1–2 LD plus freeze dryer) and gamma irradiated with 27 kGy dose (BRIT, Mumbai) to yield a sterile powder. This powder was then mixed into a base solvent to a desired concentration calculated in mg/ml. The type of solvent used depended on the test as shown in Table 1.

**Table 1:** Preparation of secretome formulation (SF) for in vitro and in vivo study.

Type of test	Sterile freeze dried powder used in the following base solvents
2D culture in vitro	Chemically defined medium of TNBCs
3D culture in vitro	Chemically defined medium of TNBC spheroids—Mamocult medium
In vivo nude mice	Ringers lactate saline

### Characterisation of secretome formulation

- Field Emission scanning electron microscope (FESEM) and imaging- Freeze dried powder of chemically defined MSC specific medium, referred to here as freeze dried control and freeze dried secretome was loaded onto the stub for determining size of the particle components and images were acquired in Jeol JSM 7610F (JEOL, Japan).
- Protein estimation using Bradford assay and SDS -Polyacrylamide Gel Electrophoresis (SDS-PAGE)—Proteins were precipitated using methanol precipitation (1:3, sample: methanol), air dried and quantified by Bradford method. 100 µg proteins from freeze dried stem cell secretome and freeze dried control precipitates were mixed with Laemmli Buffer along with 1 × protease phosphatase inhibitor (5% V/V) (Cell signalling, USA), reduced at 95 °C for 5 min in dry bath. Reduced samples were separated using SDS PAGE (BioRad, USA) at 90 V. Gel was stained using Commassie brilliant blue and image was scanned.
- Quantification of RNA—Total RNA was extracted from freeze dried stem cell secretome and control using RNeasy kit (Qiagen, Germany) and quantified using Qubit Fluorometer (Thermofisher, USA).
- Quantification of Minerals and Glucose: Stock concentrations of 100 mg/ml of freeze dried stem cell secretome and control were prepared in Ringers lactate saline (RLS). Urea, calcium, phosphorus, magnesium, iron and potassium and glucose were quantified using specific biochemical methods (Roche Cobas, Switzerland).
- X-ray crystallography—freeze dried stem cell secretome and control powder diffractions were analysed using XPert Pro diffractometer (Panalytical, Netherlands) and graphs were plotted using Origin software (version 6.0, USA, https://www.originlab.com/).
- Quantification of lipids using Fourier transform infrared spectroscopy (FTIR) analysis: Supernatant obtained after protein precipitation of freeze dried stem cell secretome and control were mixed with tenfold volume of sterile water, frozen at -80 °C and freeze dried (Fisher Alpha, Germany). Freeze dried samples (2 mg) were blended well with potassium bromide (178 mg) and pellets of 2 mm thickness were prepared. Spectra were collected in FTIR (Shimadzu, Japan), normalized and base line corrected using Lab solutions software and compared with spectra of lecithin as reference. Graphs were plotted using Origin software (version 6.0, USA, https://www.originlab.com/).

### Breast cancer cells 2D culture, treatment with SF and MTT assay

MDA-MB-231 (5 × 10^3^ cells/well) were seeded in 96-well plates, incubated overnight, washed with PBS and treated with SF with the following controls: CM, and various concentrations of freeze dried chemically defined medium of MSCs. Toxicity of various concentrations of paclitaxel (1 nM to 100 µM) prepared in CDM MDA were also evaluated. For studies involving paclitaxel and SF, four concentrations of paclitaxel—0.17 nM, 1.6 µM, and 1.7 µM were used. The 1.6 and 1.7 µM represented the blood concentration of paclitaxel taken at an oral dose of 175 mg/m^2^ and an oral bioavailability of 6%. They were not intentionally separated but one set was found to be 1.6 and the other at a slightly different value of 1.7 µM and hence the data was shown separately. The following combinations were used: 1.6 µM paclitaxel combined with SF 50 mg/ml, 1.6 µM, and 0.17 nM paclitaxel along with SF 70 mg/ml. 5 mg/ml MTT (Sigma-Aldrich, USA) was added after 48 h. The absorbance values were measured using microplate reader (BioTek Synergy, USA) and relative viability was calculated using the formula absorbance of treatment/absorbance of control.

### Apoptosis assay

3.9 × 10^5^ cells were seeded and incubated in T25 flasks similar to MTT assay. After 48 h cells treated with SF (in mg/ml) were stained with Annexin V/PI kit (Invitrogen, USA), kept on ice and analysed using flow cytometer (BD Canto, USA).

### Morphological analysis and live dead staining

SF treated cells (method similar to apoptosis assay) were stained with calcein, ethidium homo dimer live dead assay kit (Thermo Fisher, USA). Morphological changes and live dead cells were imaged using NIS elements software (https://www.microscope.healthcare.nikon.com/products/software/nis-elements) at 10× magnification with green and red filters (Nikon, Eclipse 2000E) and processed in Image J software (https://imagej.nih.gov/ij/download.html) for background correction.

### Breast cancer stem cell, MDR1 and PDL1 analysis using flow cytometry

SF-treated cells (method similar to apoptosis assay), were incubated with antibodies specific to CD44 conjugated with PE and CD24 conjugated with FITC, multi drug resistance protein1 (MDR1) and programmed death ligand protein (PD-L1) conjugated with PE (BD, USA) in dark at room temperature for 10–20 min and analysed using BD Canto flow cytometer.

### In vivo studies

Swiss nude mice were obtained from the Indian Institute of Science, Bangalore. All animal protocols were approved by the Institutional Animal Ethics Committee at Amrita Institute of Medical sciences and Research Centre based on ARRIVE guidelines. All animals were housed in the isolated nude mice facility under specific pathogen-free conditions and maintained with sterile air, food and water throughout the study. All animal handling procedures and experiments were done under aseptic conditions in the laminar air flow until the day of euthanasia in accordance with the basic guidelines and regulations. MDA-MB-231 cell line xenografts were developed and tumor was induced in all grouped animals. In brief, 1 × 10^6^ cells suspended in 100 µl (1:1) chemically defined media: Matrigel stored on ice were subcutaneously injected into upper flank region of right hind limb of 8–12 weeks old female nude mice to ensure good blood circulation for tumor growth. All mice were randomly grouped into three (control, dose I and dose II) and sample size of n = 6 was determined by power analysis using G-Power software (https://www.gpower.hhu.de/). Body weight and activity of animals were monitored and noted everyday post tumor induction.

100 µl of two concentrations (50 mg/ml (Dose I) and 100 mg/ml (Dose II)) along with saline control was used as vehicle control. Palpable tumors were carefully monitored and measured for length and width using digital Vernier caliper. Tumor volumes were calculated using the formula(1/2 Length × width^2^) and intra-tumor injections were administered for 21 days into an initial tumor volume of 1 mm^3^ based on volumetric calculation corresponding to 100 µl. Animals were first anaesthetized with 2–2.5% isoflurane and 80% oxygen for all subcutaneous and intra tumor injections. T1 and T2 weighted MRI data of animals from each group were collected using small animal MRI scanner (Bruker, USA) before euthanasia.

Animals were euthanized by overdose ketamine IP injections. Blood was collected, centrifuged at 1,000 g for 5 min at room temperature and total serum calcium quantified (Roche Cobas 8000, Switzerland). Tumors dissected were fixed in NBF for histopathology study and a quarter of the dissected tissue was homogenised, digested with 0.3% collagenase and strained using 0.45 µm sized cell strainer. Strained cells were stained similar to method specified for CD44^+^/CD24^−^, MDR1 and PD-L1 flow cytometry analysis. For treatment samples that were analysed in triplicates, cells were pooled from excised tissue of 2 animals out of 6 in Dose 1 and 2 groups.

### 3D cultured TNBC spheroids, treatment with SF and toxicity assays

MDA-MB-231 (5 × 10^3^ cells) was 3D cultured in ultra-low attachment plates (Corning, USA) in Mammocult medium (Stem Cell Technologies, Canada). Spheroids were centrifuged at 270 g for 5 min at room temperature and treated with SF, paclitaxel and combination of both for 48 h. Toxicity of various concentrations of SF (in mg/ml) on 3D spheroids was evaluated using MTT assay. For studies involving SF and paclitaxel combinations 3D cell titre glow assay (Promega, USA) was performed and luminescence was measured in white-bottom plates (Thermo Fisher, USA) in microplate reader (BioTek Synergy, USA). Live cell imaging was performed using confocal microscope (Leica, TCS SP5 II, Germany) after staining with live dead assay kit (Thermo Fisher, USA). For all studies involving spheroids, MDA-MB-231 spheroids in Mammocult medium was used as control. SF 70 mg/ml, paclitaxel 1.7 µM and combinations were prepared in Mammocult medium for treating spheroids.

### Statistical analysis

Statistical analysis was performed using Graph Pad Prism V.5 (Graph Pad Software, USA, https://www.graphpad.com/scientific-software/prism/). All graphs were plotted with mean ± SD values and student’s t-test was performed to analyse parametric paired data. Differences were considered significant when *p* value < 0.05 and is represented by (*). All experiments were a minimum of three independent replicates and otherwise mentioned along with the data description.

## Supplementary Information

Supplementary Information.
