# Author Correction: Adipose derived mesenchymal stem cell secretome formulation as a biotherapeutic to inhibit growth of drug resistant triple negative breast cancer

**DOI:** 10.1038/s41598-023-38226-2

**Published:** 2023-07-07

**Authors:** Ragima Nadesh, Krishnakumar N. Menon, Lalitha Biswas, Ullas Mony, K. Subramania Iyer, Sundeep Vijayaraghavan, Ajit Nambiar, Shantikumar Nair

**Affiliations:** 1 Amrita Centre for Nanosciences and Molecular Medicine, Amrita Vishwa Vidyapeetham, Kochi, Kerala 682041 India; 2 Amrita Institute of Medical Sciences and Research Centre, Amrita Vishwa Vidyapeetham, Kochi, Kerala 682041 India

Correction to: *Scientific Reports* 10.1038/s41598-021-01878-z, published online 06 December 2021

The original version of this Article contained errors in Figure 1 (d) label, where SI units were incorrect.

“Calcium (in mg/ml)”

now reads:

“Calcium (in mg/dl)”

“Phosphorus (in mg/ml)”

now reads:

“Phosphorus (in mg/dl)”

“Iron in (mg/ml)”

now reads:

“Iron in (ug/dl)”

And in Figure 1 (e) x-axis label,

“(Glucose in mg/ml)”

now reads:

“(Glucose in mg/dl)”

Additionally, the Article contained an error in Figure 2 (g) y-axis label, where

“Relative cell viability”

now reads:

“Percent of cells”

The original Figure 1 and 2 and accompanying legends appear below.

**Figure 1:**
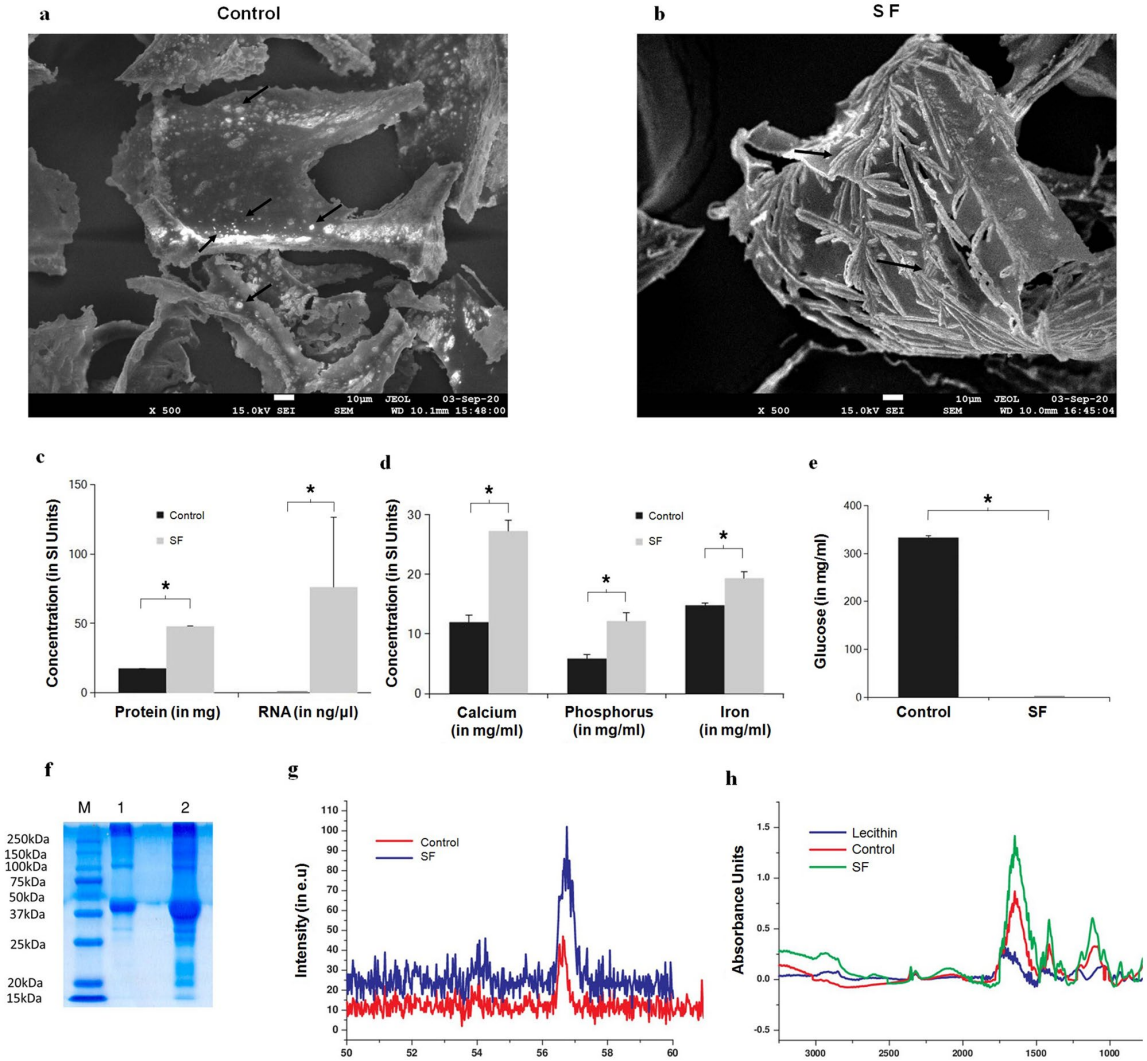
Characterization of freeze dried adipose derived stem cell secretome. (**a**) FESEM image of freeze dried medium alone (that is without secretome), black arrows shows crystalline particles, (**b**) FESEM image of freeze dried secretome, black arrows shows acicular crystalline flakes suggesting protein and/or RNA content in SF; (**c**) Graphical plots of protein (n = 3) and RNA (n = 3) quantified showing higher concentrations of proteins, and also higher levels of RNA in SF; (**d**) Graphical plots of minerals profiled of Calcium, phosphorus and iron again showing increased content in SF, (**e**) estimated glucose levels showing negligible glucose in SF; (**f**) Scanned Image of SDS Page (M—Marker proteins, Lane 1—control and Lane 2—freeze dried secretome) confirming more numerous and higher protein content in SF compared to control; (**g**) Graphical plots of XRD analysis with the main peak confirming higher mineral content. (**h**) Graphical plots of FTIR analysis, with the peaks indicating higher lipid levels in SF. All graphical plots are mean ± SD values and differences between control and treatment concentrations were considered statistically significant for p value < 0.05 and is represented by (*).

**Figure 2:**
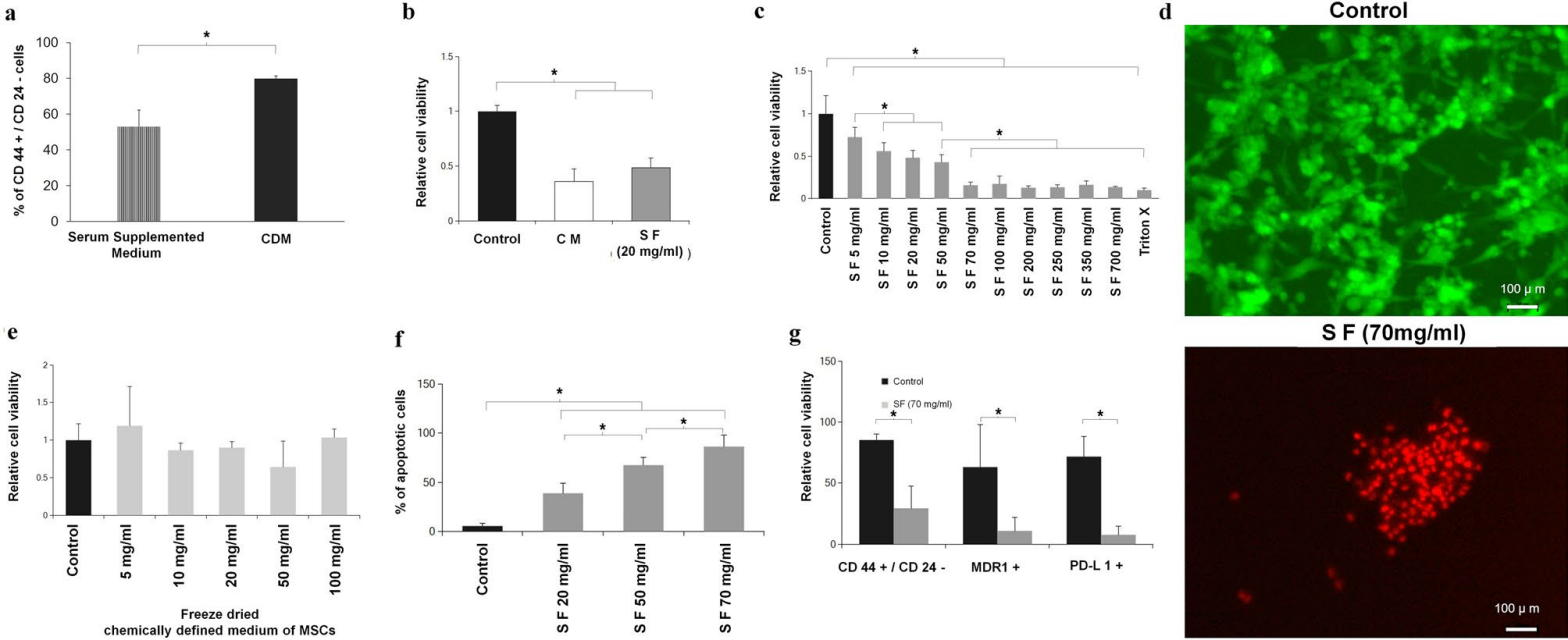
Adipose derived stem cell secretome on TNBC viability, apoptosis and molecular markers. (**a**) Flow cytometry results of TNBCs in serum supplemented medium and chemically defined medium with CD44^+^/CD24^−^ breast cancer stem cell phenotype after 48 h. The chemically defined medium increased marker concentration because of the presence of insulin, hydrocortisone and epidermal growth factor that are known to contribute to colony formation characteristic feature of cancer stem cells^51^. (**b**) Graphical plot of cell viability comparison between control (medium without MSC exposure), MSC conditioned medium (without lyophilization), and SF. SF at the low concentration of 20 mg/ml is equivalent to the MSC conditioned media results. (**c**) Dose dependent effect of SF on TNBC viability at 5, 10, 20, 50, 70, 100, 200, 250, 350 and 700 mg/ml. Above a dose of 50 mg/ml SF has substantial cell kill. (**d**) Effect of secretome free media showing that lyophilization of the media alone does not affect cell viability. Green color shows calcein binding to actin filaments in live cells. (**e**) Fluorescence image of live dead staining of TNBCs treated with 70 mg/ml SF for 48 h showing cell kill. Red color shows ethidium homodimer binding to nucleic acid in dead cells. (**f**) Apoptosis of cells treated with varying concentrations of SF for 48 h evaluated by Annexin-V–PI staining using flow cytometry showing an increase in cancer cell apoptosis. (**g**) Histogram of flow cytometry results showing the effect of 70 mg/ml SF on CD44^+^/CD24^−^ breast cancer stem cells (CSCs), multi drug resistance protein 1 (MDR1) and programmed death ligand-1(PD-L1) expressing cells. All markers are substantially decreased by SF. See text for discussion. All graphical plots are mean ± SD values and differences between control and treatment concentrations were considered statistically significant for p value < 0.05 and is represented by (*).

The original Article has been corrected.

