# Supplementary material for: Adipose derived mesenchymal stem cell secretome formulation as a biotherapeutic to inhibit growth of drug resistant triple negative breast cancer
Source: Sci Rep. 2021 Dec 6;11:23435. doi: 10.1038/s41598-021-01878-z (PMC8648896; doi:10.1038/s41598-021-01878-z)
Supplement: Supplementary file 1 — Supplementary Information. [file 41598_2021_1878_MOESM1_ESM.pdf]

Adipose derived mesenchymal stem cell secretome formulation as a biotherapeutic to inhibit growth of drug resistant triple negative breast cancer

Ragima Nadesh[1], Krishnakumar N Menon[1], Lalitha Biswas[1], Ullas Mony[1], Subramania Iyer K[2], Sundeep Vijayaraghavan[2], Ajit Nambiar[2] and Shantikumar Nair[1*]

Supplementary material with extended data of main figures

Preparation of MSC secretome for *in vitro* and *in vivo* study in detail.

Passage 3-7 MSCs were seeded in T75 flasks and when 80-90% confluent, cells were washed with PBS and incubated for 48 hours in 15 ml of chemically defined MSC specific medium (Stem-pro MSC SFM CTS, Gibco, Life technologies) supplemented with growth supplement, 1% (200 mM) glutamine (Thermo Fisher Scientific) and 1% antibiotics (Anti-Anti, Gibco, Life technologies) for preparing stem cell conditioned media. This consists of stem cell secretome and other components of defined media that were not utilised by MSCs within 48hours of incubation. The freeze dried powder is expected to contain secretome along with some other constituents. 20 mg of powder was obtained from each ml of the conditioned media.

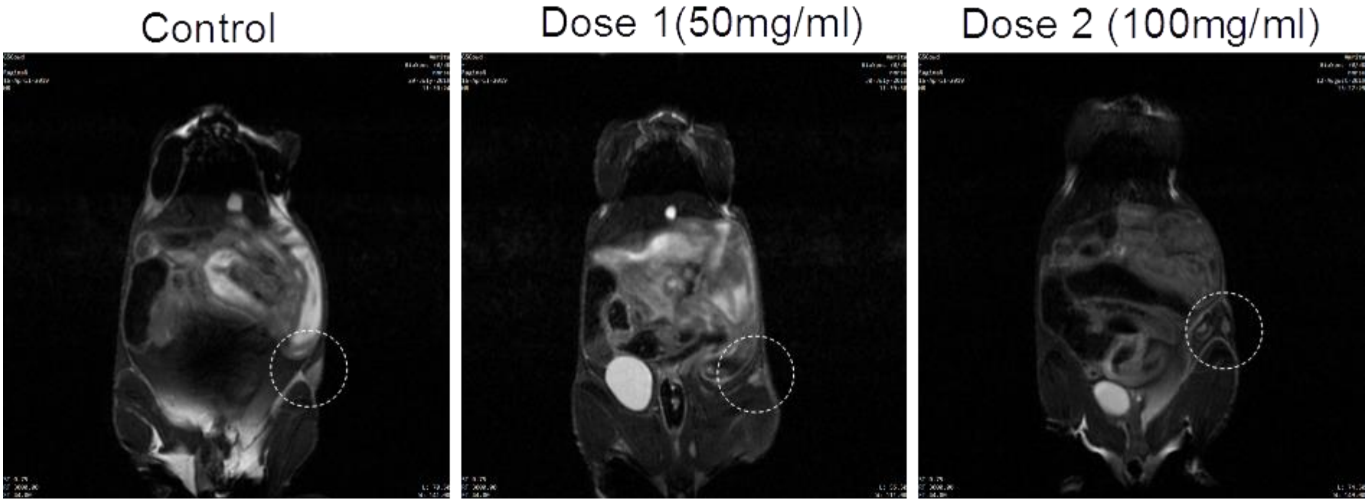

Supplementary data Figure1. Representative MRI images of animals from each group.

Quantification of Urea in SF (70 mg/ml) n=1

Urea in control (100mg) – 1.36 mg/dl

Urea in SF (100mg) – 2.83 mg/dl

Calculation of IC50 for 2D and 3D cultures

To calculate the IC50 values and their spread, the data in Fig. 2C (2D culture) and in Fig 3A (3D culture) was replotted with the concentration shown on the x-axis in log scale, see Supplementary Fig. 2A and Fig. 2B below. Then Prizm software was used to employ a non-linear regression fit to evaluate the IC 50 value and its spread as each concentration was evaluated experimentally in triplicate. The IC50 in 2D culture was 10.54 mg/ml (with a range from 6.534 to 15.29 mg/ml) and in 3D culture the *50* was 32.57 mg/ml (with a range from 27.88 to 47.54 mg/ml). The IC50 in 3D was about three times that in 2D cultures, indicating greater resistance to SF in 3D culture, which signifies the role of the cellular microenvironment on cell resistance.

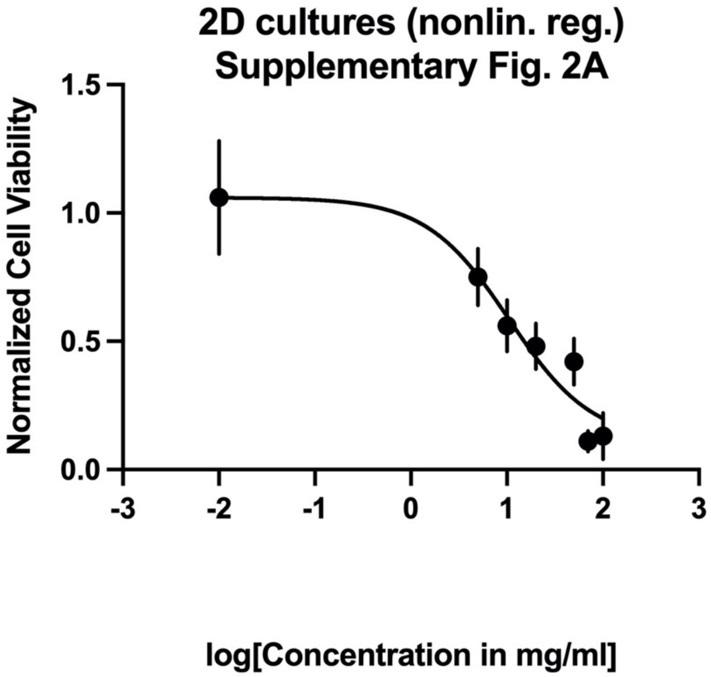

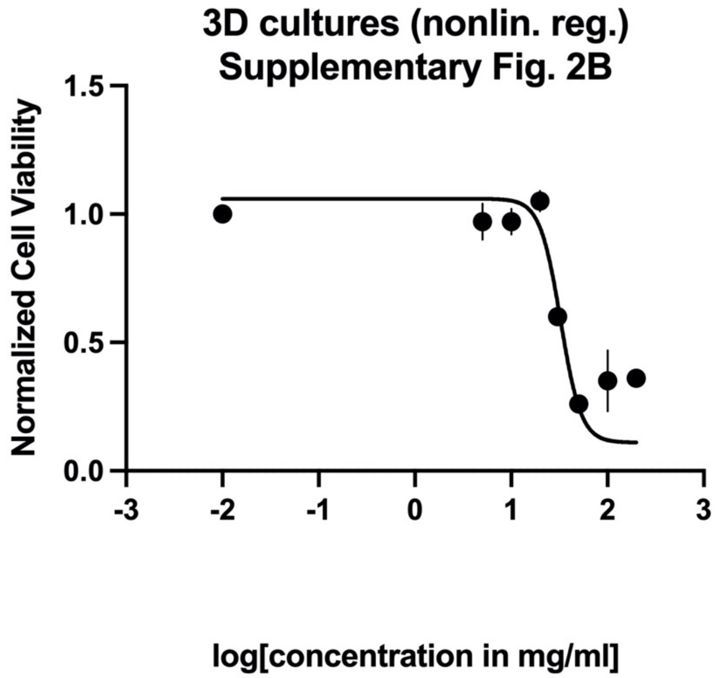
3D cultures (nonlin. reg.)
Supplementary Fig. 2B
Normalized Cell Viability
1.5
1.0
0.5
0.0
-3
-2
-1
0
1
2
3
log[concentration in mg/ml]
